# *BCL6*, *DUSP3*, and *IL6R* Are Identified as Shared Druggable Immune-Regulatory Axis in Atrial Fibrillation and Atherosclerosis Through Integrative In Silico and In Vitro Analysis

**DOI:** 10.1155/humu/7388320

**Published:** 2025-11-11

**Authors:** Haotian Zheng, Linxin Yang, Pengli Zhu, Yazhou Lin

**Affiliations:** ^1^Department of Cardiology, Shengli Clinical Medical College of Fujian Medical University, Fuzhou University Affiliated Provincial Hospital, Fujian Research Institute of Cardiovascular Diseases, Fuzhou, Fujian Province, China; ^2^Department of Ultrasound, Shengli Clinical Medical College of Fujian Medical University, Fuzhou University Affiliated Provincial Hospital, Fuzhou, Fujian Province, China; ^3^Department of Geriatric Medicine, Shengli Clinical Medical College of Fujian Medical University, Fujian Provincial Institute of Clinical Geriatrics, Fuzhou University Affiliated Provincial Hospital, Fujian Provincial Center of Geriatrics, Fuzhou, Fujian Province, China

**Keywords:** atherosclerosis, atrial fibrillation, immune-regulatory genes, integrative transcriptomic analysis, sc-RNA analysis

## Abstract

**Background:**

Atrial fibrillation (AF) and atherosclerosis (ATH) are increasingly recognized as interconnected cardiovascular conditions with shared immune and inflammatory underpinnings. However, the molecular mechanisms linking their pathogenesis remain poorly defined.

**Methods:**

A multiplatform transcriptomic analysis was conducted using publicly available microarray datasets for AF and ATH. Differentially expressed genes (DEGs) were identified using linear modeling and batch correction. A total of 29 overlapping DEGs were found between AF and ATH, from which five immune-related DEGs were identified using the ImmPort database. LASSO regression selected three genes, that is, *BCL6, DUSP3*, and *IL6R*, as optimal immune-regulatory hub genes. Functional enrichment, drug–target interaction profiling, transcriptional regulatory network modeling, immune infiltration estimation, and single-cell RNA-seq analysis were conducted using R-based pipelines, DGIdb, iRegulon, CIBERSORTx, ImmuCellAI, and CellChat. To experimentally validate their regulatory role in AF, in vitro assays were performed using angiotensin II-treated mouse cardiac fibroblasts (MCFs). Gene-specific knockdown was achieved via siRNA transfection, followed by RT-qPCR, western blotting, colony formation, wound healing, and proliferation assays using Thermo Fisher–validated kits and reagents.

**Results:**

Transcriptomic pathway enrichment revealed strong involvement of MAPK signaling, phosphatase regulation, and T cell immunomodulatory pathways. Drug–gene interaction analysis identified four immune DEGs as druggable targets. Transcription factor regulatory modeling identified nine TFs converging on *BCL6*, *DUSP3*, and *IL6R*. Immune deconvolution analysis revealed macrophage and dendritic cell enrichment in both conditions, with broader immune remodeling in AF. Single-cell RNA-seq localized *BCL6*, *DUSP3*, and *IL6R* to T cells, macrophages, and fibroblasts, with divergent intercellular signaling inferred via CellChat. In vitro, siRNA-mediated knockdown of *BCL6*, *DUSP3*, and *IL6R* in Ang II-stimulated cardiac fibroblasts significantly suppressed their expression and attenuated fibroblast activation, while *CUL4A* knockdown showed supporting effects showing the pathogenic relevance of the core immune-regulatory axis in AF-ATH.

**Conclusion:**

This integrative study identifies *BCL6*, *DUSP3*, and *IL6R* as shared immune-regulatory genes in AF and ATH, with transcriptomic and in vitro evidence supporting their pathogenic role and potential as dual-disease therapeutic targets.

## 1. Introduction

Atrial fibrillation (AF) and atherosclerosis (ATH) are among the leading causes of cardiovascular morbidity and mortality worldwide, representing two distinct yet increasingly interlinked pathologies. AF is the most common sustained cardiac arrhythmia globally, with a prevalence of over 33 million individuals and rising sharply with age and comorbid conditions. AF is characterized by irregular atrial electrical activity, which contributes to adverse clinical outcomes including thromboembolism, heart failure, and stroke [1]. ATH is the progressive accumulation of lipid and fibrotic plaque within the arterial wall, associated with the pathogenesis of coronary artery disease and ischemic cerebrovascular events [2]. Despite differences in anatomical targets, these two conditions frequently coexist and share biological pathways such as inflammation, endothelial dysfunction, and immune activation [3]. Growing experimental evidence supports that AF and ATH are not isolated diseases but may represent interconnected manifestations of systemic vascular inflammation. Epidemiological studies indicate that patients with ATH are at significantly higher risk of developing AF and vice versa [4, 5]. Histological studies of atrial tissue in AF patients often reveal infiltration of macrophages, lymphocytes, and cytokine-secreting cells similar to those observed in atherosclerotic plaques [6, 7]. Furthermore, both diseases involve remodeling of vascular and myocardial extracellular matrices, disruption of endothelial integrity, and the activation of innate and adaptive immune signaling pathways [8, 9]. However, the precise molecular mechanisms that mediate this immune-based overlap remain incompletely understood.

While recent studies have begun to explore the pathogenicity biomarkers of AF and ATH independently [10], relatively few have attempted to delineate the shared immune-regulatory landscape between them. This gap in knowledge is particularly relevant given the increasing success of immune-targeted therapies in inflammatory diseases such as rheumatoid arthritis, psoriasis, and heart failure. A more detailed understanding of common immune signatures in AF and ATH could facilitate the identification of novel biomarkers and potentially uncover shared therapeutic targets for dual-disease intervention. Transcriptomic profiling offers a powerful method to interrogate the immune microenvironment in complex diseases. Previous gene expression studies in AF have highlighted dysregulation of inflammatory cytokines, stress response pathways, and T cell-related gene networks in atrial tissue [11]. Similarly, transcriptional analysis of atherosclerotic arteries has revealed the involvement of toll-like receptors, interferon-stimulated genes, and regulatory T cell pathways in plaque progression and instability [12]. Moreover, classical bulk RNA-seq and microarray analyses often obscure the cellular origin and heterogeneity of gene expression. Advances in single-cell RNA sequencing and computational tools such as CellChat [13] and SingleR [14] have enabled high-resolution dissection of cell type–specific transcriptional states and intercellular communication networks. When integrated with immune deconvolution methods like CIBERSORTx [15] and ImmuCellAI [16], these tools offer a multidimensional view of immune dynamics at both tissue and single-cell levels. Importantly, they also allow the prioritization of functionally active genes for downstream validation and translational targeting.

In this study, we performed an integrative analysis to identify shared immune-regulatory genes between AF and ATH. Differentially expressed gene (DEG) analysis on two independent microarray datasets each for AF and ATH revealed 29 commonly dysregulated genes in both diseases. Five immune-related DEGs (IDEGs) were identified via intersection with the ImmPort immune gene reference set. These genes were further evaluated using LASSO logistic regression. To contextualize these genes within biological networks, we conducted Gene Ontology (GO) and pathway enrichment analyses using clusterProfiler, Enrichr, and Metascape. Enrichment results revealed involvement in MAPK phosphatase signaling, cytokine receptor activity, and T cell differentiation. Druggability of the candidate genes was assessed using the Drug–Gene Interaction Database (DGIdb), revealing pharmacological associations for four out of five genes. Regulatory network analysis using iRegulon identified nine transcription factors (TFs) suggesting transcriptional coregulation in immune processes across both conditions. We further evaluated immune cell type abundance using CIBERSORTx and ImmuCellAI, showing shared enrichment of macrophage and dendritic cell populations in both AF and ATH. Single-cell RNA-seq datasets were then analyzed to localize expression of hub genes and TFs across annotated cell types and to model ligand–receptor-based communication pathways. Notably, CellChat-based intercellular signaling analysis revealed dominant fibroblast–macrophage interactions in AF and endothelial–dendritic circuits in ATH.

## 2. Methodology

This study was conducted using publicly available gene expression datasets obtained from the NCBI-GEO database. No ethical approval or informed consent was required for this study. All analysis steps were implemented using reproducible and peer-reviewed bioinformatics frameworks and in vitro functional validation to ensure methodological transparency and scientific rigor.

### 2.1. Data Collection

Microarray gene expression datasets and single-cell RNA datasets were retrieved from the Gene Expression Omnibus (GEO) database to investigate transcriptomic alterations associated with AF and ATH. The selection criteria required that each dataset contain both disease and control samples derived from human tissues or blood. For AF, GSE115574 [17] and GSE41177 [18] were selected as primary training datasets. GSE115574, based on the Affymetrix GPL570 platform, contains atrial tissue samples from 30 patients with AF and 29 patients in sinus rhythm (SR), resulting in 59 arrays. GSE41177, also on the GPL570 platform, includes 32 atrial tissue samples from AF patients and six from individuals in SR. For validation, GSE79768 and GSE14975 [19] were selected. GSE79768 consists of paired left and right atrial tissues from 14 individuals with persistent AF and 12 individuals in SR. GSE14975 includes 5 left atrial tissue samples from AF patients and 5 from matched controls, totaling 10 samples derived from human atrial biopsies.

For ATH, GSE100927 [20] and GSE57691 [21] were selected as training datasets. GSE100927 includes 104 arterial tissue samples profiled using the Agilent GPL17077 platform, consisting of 69 samples from atherosclerotic vessels and 35 from nonatherosclerotic control arteries. GSE57691 comprises transcriptomic profiles derived from human aortic wall tissue collected during open surgical repair of AAA and AOD and from nonatherosclerotic organ donors as controls. A total of 68 samples were analyzed using the GPL10558 platform. For validation, GSE41571 [22] and GSE13985 [23] were selected. GSE41571 contains gene expression profiles from macrophage-rich regions of human carotid atherosclerotic plaques comparing ruptured (*n* = 5) and stable (*n* = 6) plaques based on the GPL570 platform. GSE13985 comprises five whole blood samples from patients with familial hypercholesterolemia (FH) and five matched healthy controls. For ATH sc-RNA, raw matrix files were downloaded from GEO accession GSE159677 [24] while AF scRNA-seq data were obtained from GSE255612 [25]. A summary of all datasets employed in this study is presented in Table 1.

### 2.2. Data Preprocessing

Each matrix was filtered to retain only probe identifiers with corresponding annotation records in their respective platform (GPL) files. Annotation tables were parsed to extract mappings between probe IDs and official gene symbols. For each dataset, only probes with valid gene symbol assignments were retained, and multiple probes mapping to the same gene were collapsed by retaining the probe with the highest average expression across all samples. To ensure measurement consistency across arrays, expression values were normalized using the quantile normalization method implemented in the “normalizeBetweenArrays()” function from the limma package in R (Version 2025.05.1) [26]. Batch effects were corrected using the empirical Bayes framework implemented in the ComBat() function from the sva package [27]. The resulting matrices consisted of batch-adjusted, log2-transformed, and quantile-normalized expression profiles and were used for all subsequent differential expression and integrative analyses.

### 2.3. Identification of Differentially Expressed Genes (DEGs)

To identify DEGs in AF and ATH, training datasets for AF (GSE115574, GSE41177) and ATH (GSE100927, GSE57691) were modeled using linear models (lmFit) followed by empirical Bayes moderation (eBayes) [28]. DEGs were defined based on a significance threshold of adjusted *p* value (FDR) < 0.01 and absolute log2 fold change > 0.25 [29]. The top DEGs for each dataset were filtered and intersected to obtain genes commonly dysregulated in both AF datasets and both ATH datasets. Final DEG lists from the batch-corrected, merged datasets were used for visualization. Volcano plots were constructed using ggplot2 with refinement for aesthetic similarity to published reference figures, including categorical coloring of significance of the top 60 most significant genes and harmonized layout with boxed labels. Gene name harmonization was applied using case-insensitive matching and delimiter-based splitting to account for merged gene annotations. Final DEG overlap between the two diseases was determined using the intersection of cleaned and standardized gene symbol sets.

### 2.4. Functional and Pathway Enrichment Analysis

Functional enrichment analysis of the 29 intersecting DEGs was performed using the R package clusterProfiler [30], Enrichr (https://maayanlab.cloud/Enrichr/) [31], and Metascape (https://metascape.org) [32]. GO enrichment was done with an adjusted *p* value cutoff of 0.05. Visualizations were generated using the enrichGO() and dotplot() functions. Enrichr was employed to assess pathway- and ontology-level enrichment across several curated databases. We utilized the *KEGG 2021 human*, *Reactome 2022*, and *GO biological process (BP) 2025* libraries to identify statistically overrepresented pathways and processes among the input gene list. Significance of enrichment was determined using the default Enrichr scoring method, which integrates both the *p* value from the Fisher exact test and the *z*-score derived from deviation from expected rank. Visualization of enriched terms was achieved via the Epiterb notebook. To complement these findings and investigate higher-order functional clustering and transcriptional regulation, we submitted the same 29-gene list to Metascape. Enrichment analyses included GO BP and pathway clustering based on shared gene membership and semantic similarity. A functional interaction network of enriched GO terms was generated and visualized in the form of a cluster-based network with nodes representing biological terms and edges denoting functional similarity. Additionally, TF target enrichment was performed within Metascape to identify candidate upstream regulators based on statistical overrepresentation of transcriptional targets within curated TF–target databases. To assess disease relevance, RAW Graphs 2.0 was used to generate a disease–gene association circos plot [33].

### 2.5. Identification of IDEGs

To explore shared immune gene signatures in AF and ATH, an immune-related gene reference list was obtained from the ImmPort database (https://www.immport.org/shared/home) [34]. Expression matrices for both datasets were analyzed using linear modeling (limma package) to identify DEGs with adjusted *p* value < 0.01 and |logFC| > 0.25. These DEGs were then intersected with the ImmPort immune gene list to obtain IDEGs for each disease. A total of 12 immune DEGs were found in AF and 284 in ATH. The intersection of these yielded five common IDEGs across both conditions, that is, BCL6, DUSP3, IL6R, CUL4A, and DUSP10. These common IDEGs served as candidate input features for LASSO analysis. These genes were extracted from normalized expression matrices in each disease context, and labels were assigned to represent case–control status. The glmnet package in R was employed to perform LASSO logistic regression with 10-fold cross-validation [35]. To evaluate model robustness across the regularization path, 50 bootstrap replicates per *λ* value were conducted, and mean AUC and standard deviation were calculated. To further confirm the disease relevance of the five immune-related hub genes, their expression was validated in four independent validation datasets, that is, GSE79768 and GSE14975 for AF and GSE13985 and GSE41571 for ATH. These datasets were normalized and reannotated using the Affymetrix GPL570 platform.

### 2.6. Druggability Analysis of IDEGs

To identify therapeutic candidates among the shared genes implicated in both AF and ATH, drug–gene interaction mapping was performed using the DGIdb [36]. A total of 29 DEGs that were shared between AF and ATH were previously identified through integrated transcriptomic analyses. The retrieved immune interaction dataset was filtered to retain only interactions where the gene symbol matched one of the 29 shared IDEGs. Gene–drug interactions with missing gene names or drug names were excluded. The resulting intersecting set represents AF- and ATH-associated genes that are known targets of approved or investigational drugs. The circlize R package was employed to construct a chord plot [37] wherein connections were drawn between drug names and gene targets.

### 2.7. Functional Enrichment and Regulatory Network Analysis

To elucidate the functional significance and upstream regulation of the five shared IDEGs, an integrative network analysis was conducted including GO/pathway enrichment, microRNA–mRNA interaction prediction, and TF regulation analysis. ClueGO (v2.5.10), a Cytoscape plugin, was used to perform BP and KEGG pathway enrichment of the five candidate IDEGs [38]. The analysis parameters were set as follows: GO BP and KEGG ontologies were selected with a *p* value threshold of 0.01, a two-sided hypergeometric enrichment test, and multiple testing correction using Benjamini–Hochberg false discovery rate (FDR). Redundant GO terms were merged using a 50% term overlap threshold. MicroRNA regulators targeting the five IDEGs were identified using the ENCORI database. The search was constrained to *human* datasets with a minimum threshold of ≥ 2 CLIP-Seq experimental supports and pan-cancer expression evidence [39]. The miRNA–mRNA network was visualized in Cytoscape [40]. Genes were represented as yellow rectangles and miRNAs as pink rounded nodes.

To elucidate, the upstream transcriptional regulation was predicted using the iRegulon plugin within the Cytoscape platform [41]. The analysis was conducted using the “Homo sapiens, HGNC symbols” annotation system, and the search space was gene-based, with a 20 kb region centered around the transcription start site (TSS) for both motif and track rankings. The motif collection used included 10,000 position weight matrices (PWMs, 9713 total), and the track collection incorporated 1120 ChIP-seq tracks derived from ENCODE raw signals. To ensure high-confidence TF predictions, the minimum normalized enrichment score (NES) was set to 4.0. The recovery step included a rank threshold of 5000, a ROC threshold for AUC calculation of 0.03, and a stringent maximum FDR on motif similarity of 0.001. TFs were only considered if they had direct orthology (minimum identity threshold set to 0.0). Based on these parameters, iRegulon identified TFs predicted to regulate two or more of the five IDEGs through enriched binding motifs and ChIP-seq tracks.

The expression level of identified TFs was validated across independent validation datasets. Probe annotation was standardized using the GPL570 platform file, and multimapped probe identifiers were resolved by separating multiple gene symbols and retaining only nonempty entries. For each dataset, raw expression values were extracted from series matrix files using a unified parser and log2 normalized where required. Group labels were defined based on the original sample metadata: for GSE79768, samples were labeled as “AF” and “SR”; for GSE14975 as “AF” and “control”; for GSE13985 as “FH” and “healthy”; and for GSE41571 as “ruptured” and “stable” plaques. Violin plots combined with dot jitter overlays and crossbar mean summaries were generated using ggplot2.

### 2.8. Immune Infiltration Analysis

To quantify the composition of immune cell types in AF and ATH, we utilized CIBERSORTx (https://cibersortx.stanford.edu/) [42] and ImmuCellAI (http://bioinfo.life.hust.edu.cn/ImmuCellAI) [43]. For each disease, a curated dataset was generated by combining raw expression matrices from both test datasets for AF and ATH. Upon completion of the deconvolution, CIBERSORTx returned a proportion matrix with estimated relative fractions of 22 immune cell types for each sample. Since the output does not contain class labels, group annotation was performed manually by mapping GSM sample identifiers to their respective clinical categories using GEO metadata for each study. Specifically, AF and SR labels were derived from sample descriptions provided in GSE115574 and GSE41177, while ATH and control assignments were extracted from GSE100927 and GSE57691. The immune cell proportion data were filtered to exclude samples labeled as “unknown” and reshaped using the reshape2 package. Group-wise differences in immune cell abundance between cases and controls were evaluated using unpaired two-tailed Student's *t*-tests across all 22 immune cell types. *p* values were adjusted for multiple testing using the FDR method. The significance level for FDR-adjusted *p* values was set at 0.05.

To complement the immune cell deconvolution performed using CIBERSORTx, we employed the ImmuCellAI algorithm to predict the abundance of 24 immune cell types with a particular emphasis on T cell subsets in AF and ATH samples. Input datasets for this analysis comprised gene expression matrices obtained from previously preprocessed and batch-corrected microarray data for both diseases. “Immune cell abundance in groups” analysis mode under the “Microarray” data type setting was selected using ImmuCellAI's built-in statistical module. The tool returned relative infiltration scores for each immune cell type across all samples. Group-wise differences in immune cell abundance between cases and controls were evaluated using unpaired two-tailed Student's *t*-tests across all immune cell types. *p* values were adjusted for multiple testing using the FDR method. The significance level for FDR-adjusted *p* values was set at 0.05 [16].

### 2.9. Single-Cell-RNA Seq Analysis and Cell–Cell Communication Modeling

Single-cell transcriptomic data for AF and ATH were analyzed to dissect immune-regulatory dynamics at the cellular level. Seurat objects of both datasets were created with quality control filters applied: cells with fewer than 200 detected genes or greater than 10% mitochondrial gene expression were excluded [44]. Data normalization was performed, followed by the identification of 3000 highly variable features using the FindVariableFeatures() function with the “vst” method. Dimensionality reduction was achieved using principal component analysis and uniform manifold approximation and projection over the top 20 principal components. Graph-based clustering was implemented using FindNeighbors() and FindClusters() at a resolution of 0.5 [45].

Cell type annotation was conducted with SingleR, using the “HumanPrimaryCellAtlasData” from the celldex package as reference [46]. Seurat objects were converted to “SingleCellExperiment” format, and predicted cell type labels were appended for visualization using DimPlot. To validate the expression of prioritized immune genes, violin plots were generated for five hub genes and nine TFs. Additionally, immune activation was assessed using a composite interferon-alpha/gamma gene signature comprising 29 genes scored using “AddModuleScore()”. Cellular composition was quantified to analyze the distribution of annotated cell types in each disease. For intercellular communication modeling, the CellChat package was used [13]. Normalized Seurat objects, labeled by SingleR annotations, were input into CellChat as the signaling database. Functional roles of each cell population within the communication networks classified as sender, receiver, mediator, or influencer were quantified.

### 2.10. Cell Culture and Angiotensin II (Ang II) Treatment

Primary mouse cardiac fibroblasts (MCFs) were purchased from Stemcell Biotechnology Co. Ltd. (Cat. No. STM-CE-3303, Shanghai, China; https://www.stemrecell.com/primary-cell-rat-fibroblast/cardiacmuscle.html). Cells were cultured in DMEM supplemented with 10% fetal bovine serum (FBS, Thermo Fisher) and 1% penicillin–streptomycin and maintained at 37°C in a humidified atmosphere with 5% CO_2_. To mimic the AF-like profibrotic microenvironment, cells were treated with Ang II (Sigma-Aldrich) at a concentration of 1 *μ*M for 24 h prior to downstream applications.

### 2.11. siRNA Transfection

MCFs were transfected with siRNAs targeting *BCL6*, *DUSP3*, *IL6R*, and *CUL4A* (Thermo Fisher) using Lipofectamine RNAiMAX Transfection Reagent (Invitrogen) according to the manufacturer's protocol. A nontargeting siRNA served as a negative control (si-NC). Cells were harvested for RNA, protein, and functional assays 48 h posttransfection.

### 2.12. Quantitative Real-Time PCR (RT-qPCR)

Total RNA was extracted using TRIzol Reagent (Thermo Fisher) and reverse-transcribed into cDNA using the High-Capacity cDNA Reverse Transcription Kit (Applied Biosystems). RT-qPCR was conducted using PowerUp SYBR Green Master Mix (Applied Biosystems) on a QuantStudio 5 system. Relative gene expression was calculated using the 2^-*ΔΔ*Ct^ method, normalized to *GAPDH*. The following primers were used for amplification purpose: *GAPDH*-F 5⁣′-ACCCACTCCTCCACCTTTGAC-3⁣′, *GAPDH*-R 5⁣′-CTGTTGCTGTAGCCAAATTCG-3⁣′; *BCL6*-F: 5⁣′-CATGCAGAGATGTGCCTCCACA-3⁣′, *BCL6*-R: 5⁣′-TCAGAGAAGCGGCAGTCACACT-3⁣′; *DUSP3*-F: 5⁣′-TGCCGACTTCATTGACCAGGCT-3⁣′, *DUSP3*-R: 5⁣′-CGTCCATCTTCTGCCGCATCAT-3⁣′; *IL6R*-F: 5⁣′-GACTGTGCACTTGCTGGTGGAT-3⁣′, *IL6R*-R: 5⁣′-ACTTCCTCACCAAGAGCACAGC-3⁣′; *CUL4A*-F: 5⁣′-GAATGAGCGGTTCGTCAACCTG-3⁣′, *CUL4A*-R: 5⁣′-CTGTGGCTTCTTTGTTGCCTGC-3⁣′.

### 2.13. Western Blotting

Protein was extracted using RIPA lysis buffer (Thermo Fisher) supplemented with protease/phosphatase inhibitors. Equal amounts of protein were resolved by SDS-PAGE and transferred to PVDF membranes. Membranes were blocked in 5% BSA and incubated overnight with primary antibodies against *BCL6*, *DUSP3*, *IL6R*, and *CUL4A* (all from Cell Signaling Technology), followed by HRP-conjugated secondary antibodies. Detection was performed using SuperSignal West Pico PLUS Chemiluminescent Substrate (Thermo Fisher) and imaged using a ChemiDoc MP system (Bio-Rad). *GAPDH* served as the loading control.

### 2.14. Cell Proliferation Assay

Proliferation was assessed using the Cell Counting Kit-8 (CCK-8, Thermo Fisher). After transfection, cells were seeded in 96-well plates and incubated with CCK-8 reagent for 2 h. Absorbance was measured at 450 nm using a microplate reader.

### 2.15. Colony Formation Assay

Cells were seeded into six-well plates (1000 cells/well) and allowed to grow for 10–14 days. Colonies were fixed with 4% paraformaldehyde and stained with 0.5% crystal violet. Colonies containing > 40 cells were counted manually in ImageJ.

### 2.16. Wound Healing Assay

Cells were grown to confluency in six-well plates. A sterile 200-*μ*L pipette tip was used to scratch the monolayer, and detached cells were removed with PBS. Cells were cultured in serum-free medium, and images were captured at 0 and 24 h using a phase-contrast microscope. Wound closure percentage was quantified using ImageJ software.

### 2.17. Statistical Analysis

All experiments were performed in triplicate. Data are presented as mean ± standard error of the mean (SEM). Statistical comparisons were made using one-way ANOVA followed by Tukey's multiple comparisons test. A *p* value < 0.05 was considered statistically significant.

## 3. Results

### 3.1. Identification of DEGs

**Figure 1:**
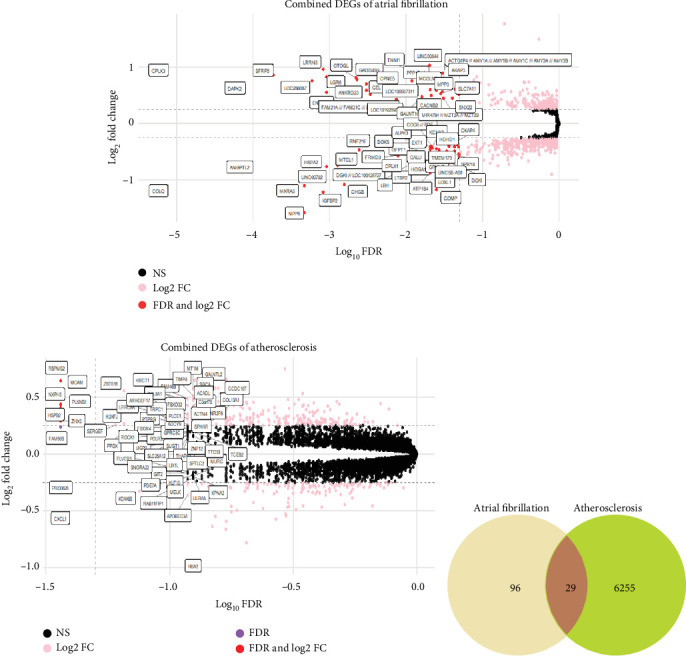
Differentially expressed genes (DEGs) in AF and ATH. (a) Volcano plot showing the distribution of DEGs in combined AF datasets. Genes with both statistically significant FDR and absolute log2 fold change > 0.25 are highlighted. (b) Volcano plot of DEGs in combined ATH datasets, illustrating widespread statistical significance with modest expression shifts. (c) Venn diagram indicating the number of unique and overlapping DEGs between AF and ATH, with 29 genes found to be commonly dysregulated.

In DEG analysis of the two AF datasets, a significant number of genes demonstrated consistent dysregulation. Specifically, 67 top DEGs were identified in the combined AF data while 5446 DEGs were identified from the combined ATH data. Volcano plots for both disease profiles revealed distinct gene expression patterns. In the AF profile, many genes including *ACTG1P4*, *ANKRD23*, *FAM21A*, and *DAPK2* were among the most significantly dysregulated with clear demarcation along both fold change and statistical significance axes (Figure 1a). Similarly, the ATH volcano plot highlighted prominent genes such as NXPH3, MCAM, *ARHGEF1*, and *ZNF12* with a dense core of statistically enriched but modestly shifted genes (Figure 1b). Upon intersection of the cleaned DEG lists, a total of 29 genes were found to be commonly dysregulated in both AF and ATH datasets. These included genes such as *CLNS1A*, *UGP2*, *RNF216*, *PHGDH*, *CKAP4*, and *IL6R*, many of which are known to participate in inflammatory pathways, oxidative stress, and endothelial dysfunction. The DEG overlap is visualized in a Venn diagram (Figure 1c) showing 96 AF-specific, 6255 ATH-specific, and 29 shared genes.

### 3.2. Pathway Enrichment Analysis

**Figure 2:**
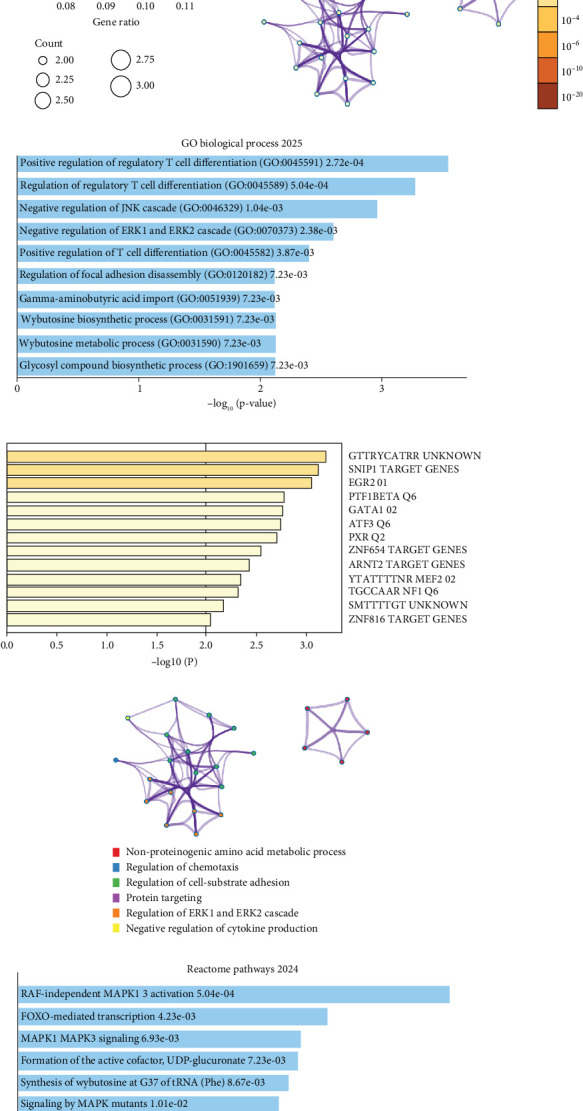
Functional and pathway enrichment analysis of the 29 intersecting DEGs in AF and ATH. (a, b) GO molecular function enrichment plots showing significant phosphatase-related activity terms. (c) Metascape-generated term interaction network depicting clusters of functionally related pathways. (d) Enrichr GO biological process 2025 results highlighting immune and signaling terms. (e) Transcription factor enrichment showing top predicted regulators of the shared DEGs. (f) ClueGO pathway cluster visualization with color-coded biological processes such as cytokine regulation and ERK signaling. (g) Reactome pathway 2024 enrichment plot illustrating MAPK and IL signaling relevance. (h) KEGG 2021 human pathway analysis indicating involvement of glycometabolic pathways.

Initial GO enrichment for molecular function revealed significant overrepresentation of phosphatase activities among the shared DEGs (Figure 2a,b). Specifically, “protein tyrosine phosphatase activity,” “MAP kinase phosphatase activity,” and “protein tyrosine/serine/threonine phosphatase activity” were the most enriched functions, which suggests deregulated dephosphorylation and MAPK signaling as shared mechanisms in AF and ATH. Metascape-based network analysis highlighted coherent clusters centered on immune regulation, signal transduction, and stress response (Figure 2c). GO BP analysis from Enrichr further emphasized T cell differentiation, *JNK/ERK* cascade modulation, and focal adhesion disassembly (Figure 2d).  Table 2 highlights nominally enriched immune-modulatory processes (*p* < 0.01, *q* > 0.05), including positive regulation of T cell differentiation, regulation of JNK and ERK cascades, and focal adhesion disassembly, driven by recurrent genes *DUSP10*, *DUSP3*, and *BCL6*. TF target enrichment (Figure 2e) identified regulatory influence from *EGR2*, *GATA1*, *PTF1*, *BETA*, and *MEF2* TFs, which are known to mediate cardiovascular remodeling, endothelial activation, and systemic inflammation. ClueGO term mapping and clustering illustrated key biological themes such as “negative regulation of ERK1/ERK2 cascade,” “chemotaxis,” “regulation of cell-substrate adhesion,” and “cytokine production” (Figure 2f). Reactome pathway analysis (Figure 2g) revealed critical enrichment of “RAF-independent MAPK1/3 activation,” “MAPK signaling by MAPK mutants,” and “IL-4/IL-13 signaling.” These pathways are consistent with immune-endothelial cross-talk and maladaptive remodeling. In parallel, KEGG pathway analysis identified significant involvement of “terpenoid backbone biosynthesis,” “galactose metabolism,” and “glycosyl compound biosynthesis” which suggests convergence on metabolic reprogramming and glycosylation-related pathways (Figure 2h).

**Figure 3:**
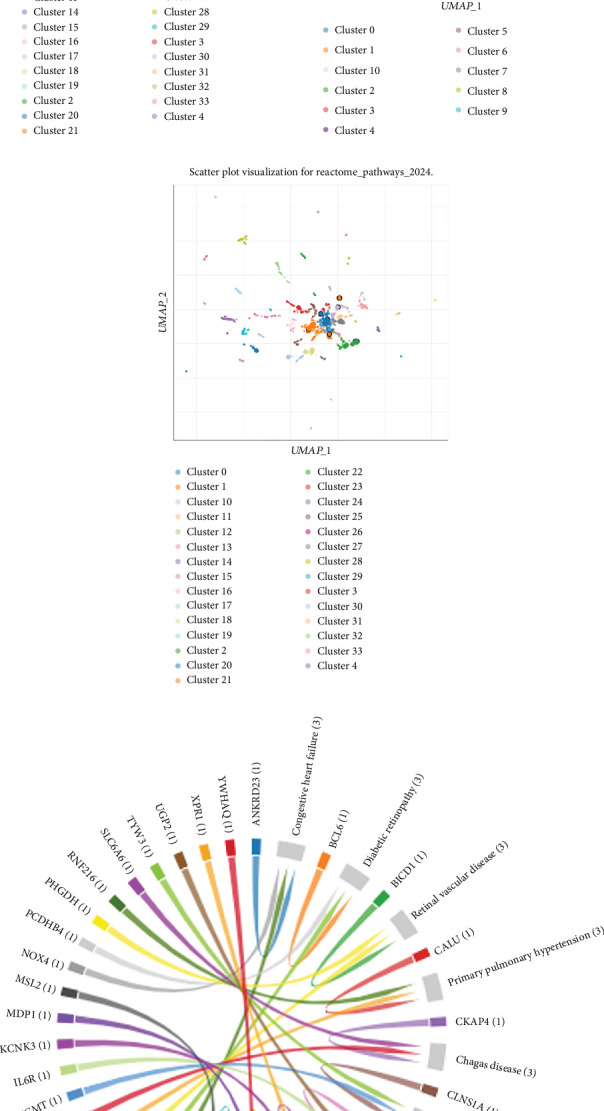
Cross-platform visualization of functional enrichment across GO, KEGG, and Reactome databases. (a–c) Enrichr-based scatterplots showing clustering of significantly enriched terms from GO biological process 2025, KEGG 2021 human, and Reactome pathway 2024, respectively. (d) Disease–gene association circos plot linking intersecting DEGs with cardiovascular and metabolic disorders.

Enrichr-derived scatterplots of the top three pathway categories demonstrated consistent clustering of enriched terms, with immune-metabolic modules forming discrete data clusters (Figures 3a, 3b, and 3c). Additionally, a disease–gene association circos plot generated using RAW Graphs 2.0 (Figure 3d) showed tight linkage of hub DEGs such as *DAPK2*, *DUSP3*, *IL6R*, and *UGP2* to cardiovascular comorbidities, including heart failure, diabetic retinopathy, cardiomyopathy, and pulmonary hypertension.

### 3.3. Identification of Immune-Related Hub Genes

**Figure 4:**
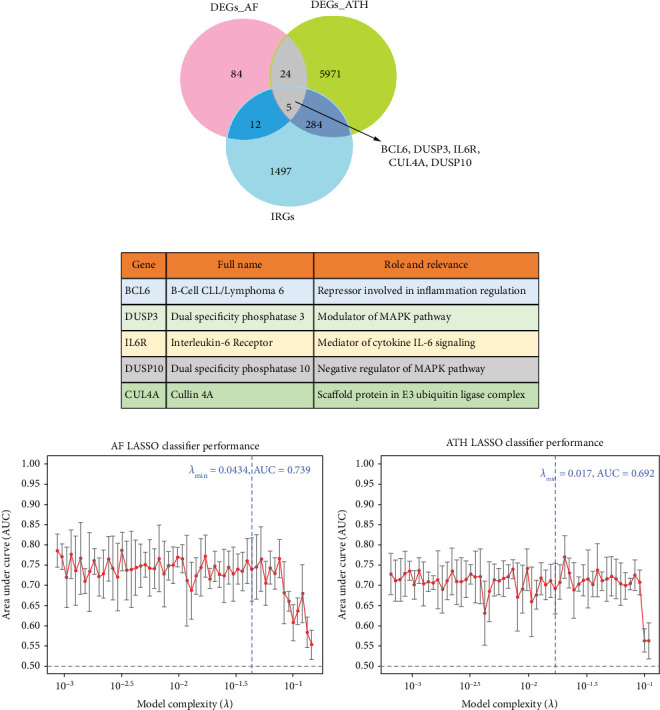
Identification and classification performance of common immune-related DEGs across AF and ATH. (a) Venn diagram illustrating the intersection of DEGs from AF, ATH, and immune-related gene (IRG) datasets. (b) Functional roles of the five intersecting immune DEGs annotated with full gene names and mechanistic relevance. (c, d) LASSO regression model performance for disease classification in AF and ATH. The models were constructed using the five common immune DEGs.

Initial DEG analysis identified 12 IDEGs in AF and 284 in ATH. The intersection yielded five common immune DEGs (*BCL6*, *DUSP3*, *IL6R*, *CUL4A*, and *DUSP10*) shared across both diseases (Figure 4a). Functional annotation confirmed that these genes participate in cytokine signaling, phosphatase activity, and inflammatory repression (Figure 4b). These five genes were used as input features for LASSO modeling.

**Figure 5:**
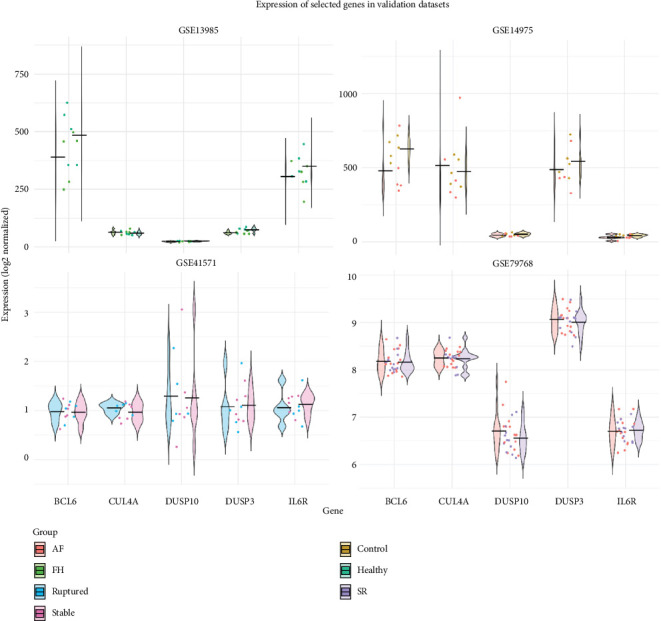
Expression validation of five immune-related hub genes in independent microarray datasets. Each subplot displays group-specific expression distributions using violin plots or scatter overlays, with group labels including AF (atrial fibrillation), SR (sinus rhythm), FH (familial hypercholesterolemia), healthy, ruptured, stable, and controls. Overall, the hub genes exhibited consistent expression patterns that distinguish disease from control samples.

LASSO regression analysis resulted in three final hub genes with nonzero coefficients at optimal *λ* in both AF and ATH, that is, *BCL6*, *DUSP3*, and *IL6R*. These genes consistently contributed to disease classification performance, which suggests their shared immune relevance across AF and ATH pathophysiology. The classifier achieved a maximum AUC of 0.739 in AF and 0.692 in ATH at optimal *λ* values of 0.0434 and 0.017, respectively (Figure 4c,d). Error bars in the performance plots reflect consistent performance stability across bootstrap iterations. The expression profiles in validation microarray datasets demonstrated consistent disease-associated patterns across both conditions. Notably, *BCL6*, *DUSP3*, and *IL6R* (LASSO-selected genes) showed distinct upregulation or downregulation trends between case and control groups (Figure 5). These results show the potential diagnostic and mechanistic value of the identified immune hub genes in both AF and ATH.

### 3.4. Drug–Target Analysis of Shared Immune Genes

**Figure 6:**
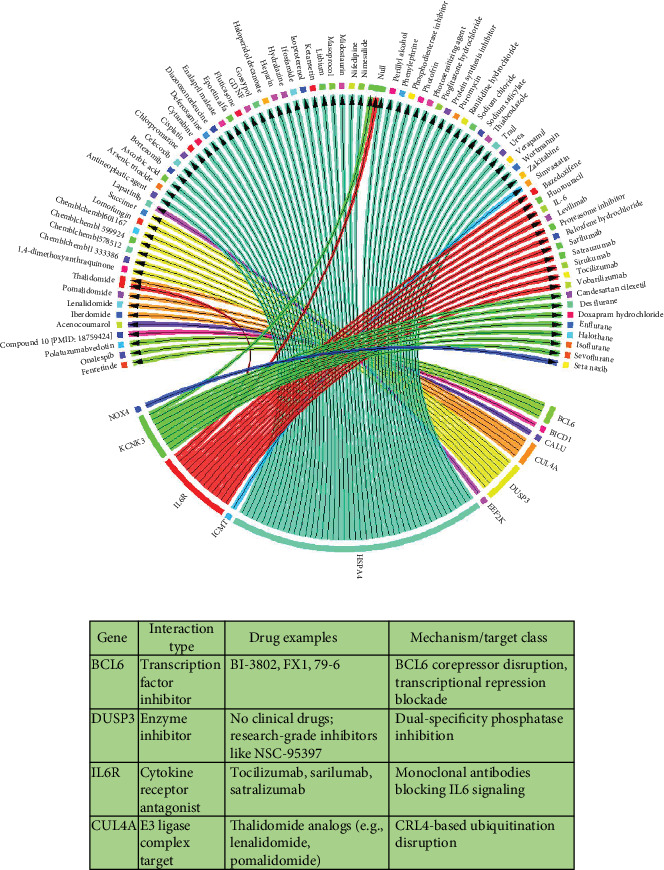
Drug–target interaction analysis of shared immune hub genes in AF and ATH. (a) Circular chord diagram representing validated drug–gene interactions identified from DGIdb for the intersecting immune-related DEGs. Nodes denote genes and pharmacological agents; edges represent known or predicted interactions, color-coded by drug category. (b) Summary table of interaction types, representative drugs, and mechanisms for the four druggable immune-related hub genes.

Out of the 29 genes shared between AF and ATH, 16 were identified as druggable using data integration from the DGIdb. High-confidence interaction mapping revealed dense pharmacological connectivity among a subset of these genes, with notable enrichment for immunomodulatory and cardiovascular-relevant agents (Figure 6a). Importantly, four of the five shared immune-related hub genes (*BCL6*, *DUSP3*, *IL6R*, and *CUL4A*) were included in the druggable gene subset, including all three genes (*BCL6*, *DUSP3*, and *IL6R*) previously prioritized by LASSO modeling. Among these, *IL6R* emerged as a well-established therapeutic target, with approved monoclonal antibodies designed to antagonize IL-6 signaling [47]. *BCL6*, a transcriptional repressor involved in inflammatory regulation, was associated with several small-molecule inhibitors [48]. Although *DUSP3* does not currently have FDA-approved inhibitors, research-grade compounds such as NSC-95397 demonstrate potential for selective dual-specificity phosphatase inhibition [49]. *CUL4A* is a member of the E3 ubiquitin ligase scaffold complex which modulates CRL4-dependent ubiquitination (Figure 6b) [50].

### 3.5. Functional and Regulatory Network Analysis of Immune-Related Hub Genes

**Figure 7:**
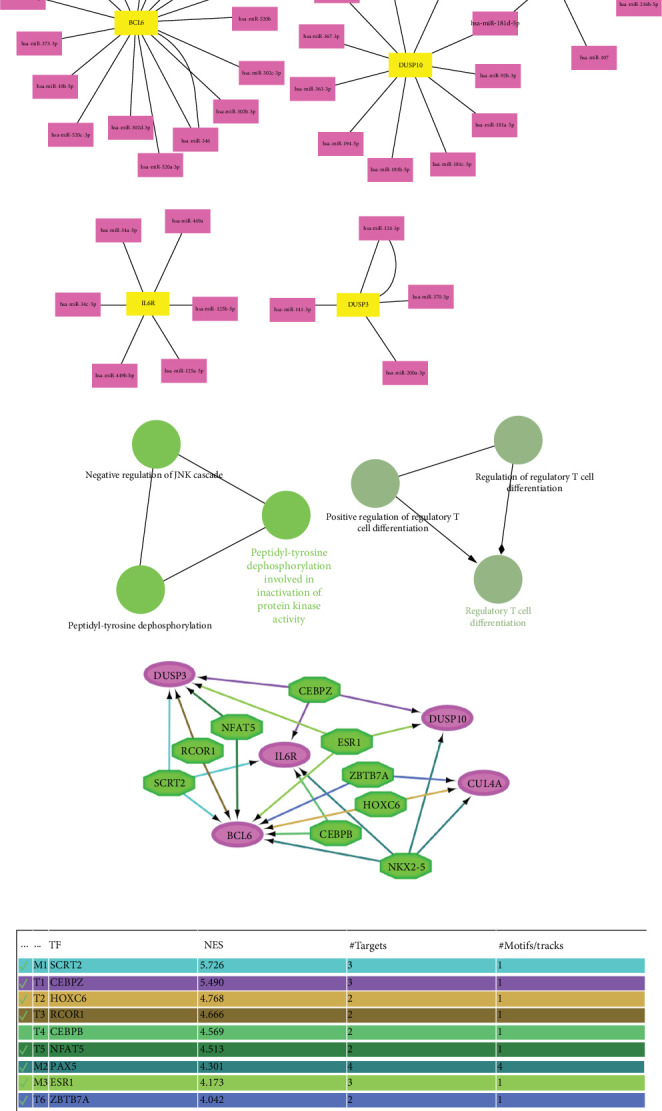
Functional and regulatory network analysis of shared immune-related hub genes in AF and ATH. (a) miRNA–gene interaction network derived from ENCORI illustrating 41 unique miRNAs targeting five immune-related DEGs. *BCL6* shows the highest miRNA interaction density. (b) ClueGO pathway analysis showing six significantly enriched GO terms related to immune signaling, kinase regulation, and T cell differentiation. (c) iRegulon-derived transcription factor network. Ten TFs with NES > 4.0 were identified as regulators of the five IDEGs. *BCL6* and *IL6R* were the most densely connected nodes, each regulated by multiple high-confidence TFs.

miRNA–gene regulatory interactions revealed 41 unique miRNAs targeting the five IDEGs (Figure 7a). *IL6R* was notably regulated by *hsa-miR-34a-5p*, *hsa-miR-125b-5p*, and *hsa-miR-449b-5p*, while *DUSP3* was targeted by *hsa-miR-124-3p*, *hsa-miR-370-3p*, and *hsa-miR-200a-3p*. *BCL6* showed the highest miRNA complexity, being targeted by more than 15 distinct miRNAs. This miRNA interaction landscape indicates intricate posttranscriptional regulation of key immune genes within both disease contexts. ClueGO analysis of the five IDEGs revealed six enriched nonredundant GO terms (Figure 7b). Key functions included “peptidyl-tyrosine dephosphorylation involved in inactivation of protein kinase activity,” “regulatory T cell differentiation,” and “negative regulation of JNK cascade.” These annotations support a mechanistic role for the IDEGs in immunological signal attenuation and T cell modulation. Importantly, the cluster specificity score indicated that 100% of input genes were recognized, and 60% were successfully annotated with GO or KEGG functional terms.

The iRegulon analysis identified a core set of TFs with strong regulatory potential over the five IDEGs, forming an integrated TF–gene interaction network (Figure 7c). A total of nine TFs passed the NES threshold and regulated at least two target genes. Among these, SCRT2 displayed the highest regulatory confidence (NES = 5.73), targeting *BCL6*, *DUSP3*, and *IL6R*. CEBPZ similarly demonstrated high NES (5.49) and also regulated three IDEGs. Other TFs included HOXC6 (NES = 4.77), RCOR1 (NES = 4.67), CEBPB (NES = 4.57), NFAT5 (NES = 4.51), and PAX5 (NES = 4.30), each targeting two or more IDEGs. Additionally, ESR1 (NES = 4.17) and ZBTB7A (NES = 4.04) were predicted with sufficient regulatory confidence and contributed to upstream convergence on *BCL6*, *CUL4A*, and *IL6R*. Visualization of the TF–gene network in Cytoscape revealed that *IL6R* and *BCL6* are the most centrally regulated genes, each receiving input from at least seven distinct TFs. *DUSP3* and *DUSP10* were moderately regulated, receiving input from four and three TFs, respectively, while *CUL4A* showed upstream association with *ZBTB7A*, *HOXC*6, and *NKX2-5*.

**Figure 8:**
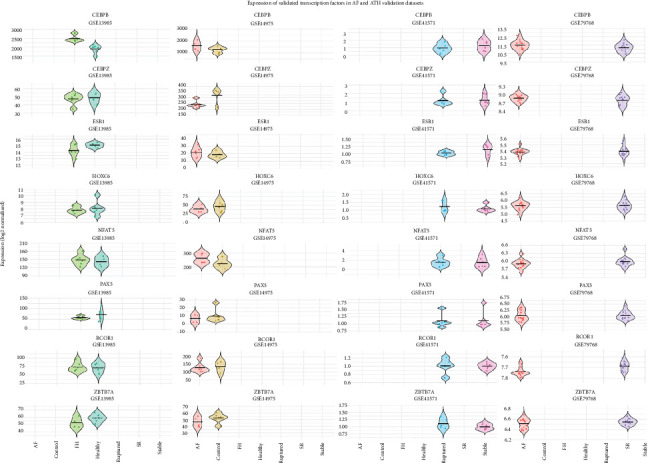
Expression validation of transcription factors in independent AF and ATH microarray datasets. Violin plots showing normalized expression of nine transcription factors across four validation datasets. Group-specific distributions (e.g., AF, SR, FH, healthy, ruptured, stable, and control) are plotted per dataset. *RCOR1* and *ZBTB7A* show stable elevation across multiple datasets while *NFAT5* and *ESR1* display contrasting regulation in inflamed versus stable tissues.

Expression validation revealed that *RCOR1* and *ZBTB7A* exhibited consistently elevated expression across AF and ATH samples in GSE79768, GSE41571, and GSE13985, suggesting their involvement as stable cross-disease regulatory candidates. In contrast, *CEBPZ* and *CEBPB* displayed elevated expression specifically in GSE13985 and GSE14975, indicating potential disease-context dependency. *NFAT5* showed marked upregulation in ruptured plaques (GSE41571) and FH samples (GSE13985), supporting its role in vascular inflammation and remodeling. Conversely, *ESR1* expression appeared reduced in stable conditions within GSE41571 and GSE79768, suggesting anti-inflammatory regulatory potential. *PAX5* and *SCRT2* demonstrated variable expression trends (Figure 8). These findings confirm the presence and regulation of key TFs across both diseases.

### 3.6. Immune Infiltration

**Figure 9:**
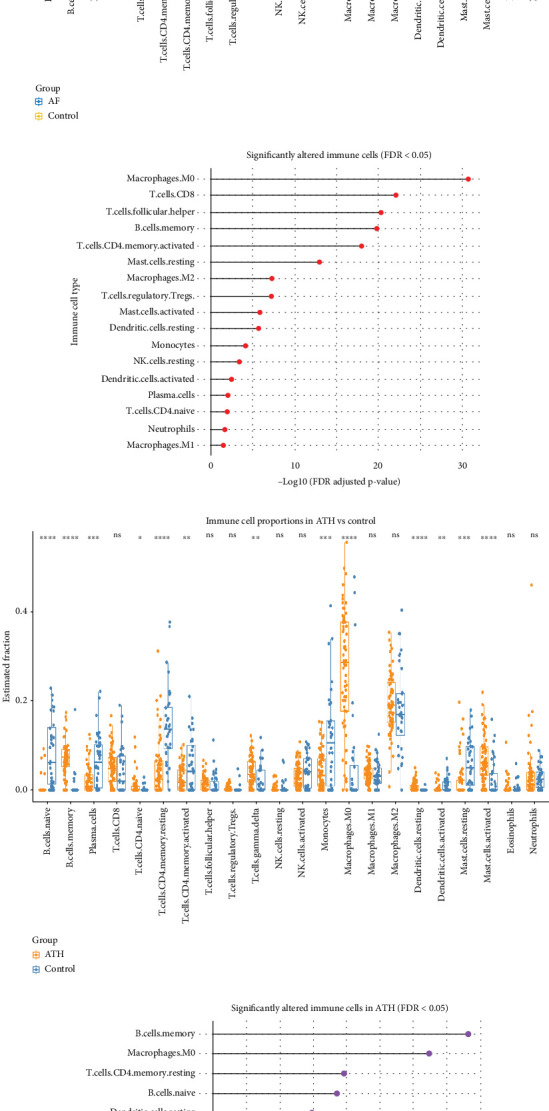
Immune cell profiling in AF and ATH using CIBERSORTx. (a) Immune cell proportions in atrial fibrillation (AF) versus sinus rhythm (SR) controls, highlighting significant alterations in CD8^+^ T cells, M1 macrophages, resting dendritic cells, and activated mast cells. (b) Statistical significance (−log_10_ FDR) across 22 immune cell types in AF; macrophages M0 demonstrated the most significant alteration. (c) Immune cell fractions in atherosclerotic arterial tissue compared to healthy controls, showing differential abundance of macrophages M0, memory B cells, and resting CD4^+^ memory T cells. (d) ATH plot summarizing significant immune shifts; macrophages M0 and memory B cells emerged as dominant contributors to immune remodeling.

Comparison of immune cell profiles between AF patients and controls revealed differential enrichment patterns across several immune compartments.  Figure 9a demonstrates distinct shifts in proportions of specific cell types in AF. CD8^+^ T cells, M1 macrophages, resting dendritic cells, and activated mast cells were all significantly altered in AF relative to SR controls (FDR-adjusted *p* < 0.05). Notably, CD8^+^ T cells were reduced in AF, whereas M1 macrophages were increased, and both mast cells and dendritic cells showed statistically meaningful shifts. The statistical summary of immune changes is illustrated in the lollipop plot (Figure 9b) which shows that 21 out of 22 immune cell types exhibited variable degrees of significance. Macrophages M0 emerged as the most profoundly altered population (−log_10_FDR > 30). Other significantly affected subsets included memory B cells, activated CD4^+^ memory T cells, and T follicular helper cells. Only a few cell types, such as resting NK cells and neutrophils, remained unchanged, suggesting selective remodeling of immune niches in AF. In ATH tissue versus healthy arterial controls (Figure 9c), significant differences were detected in memory B cells, M0 macrophages, resting CD4^+^ memory T cells, and resting dendritic cells. Among these, M0 macrophages showed the most pronounced elevation in ATH, supporting a myeloid-dominant immune profile. The lollipop plot for ATH (Figure 9d) confirmed significant enrichment in 14 out of 22 immune cell types, including dendritic cells (activated), mast cells (resting), and naïve CD4^+^ T cells.

**Figure 10:**
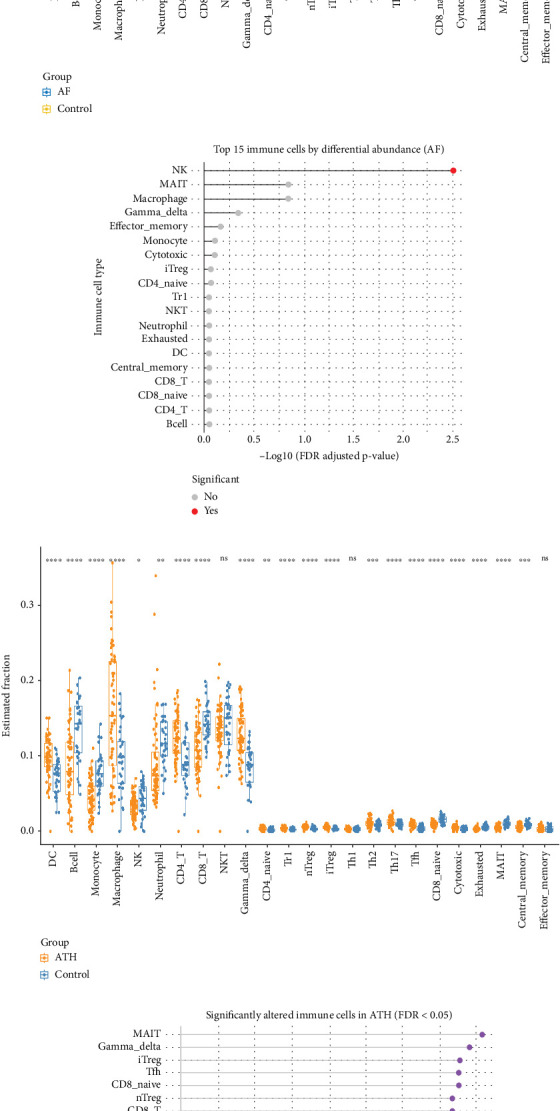
Immune cell profiling in AF and ATH using ImmuCellAI. (a) Differential immune cell abundance in AF versus control samples. Only NK cells were significantly elevated (FDR *q* < 0.05). (b) Statistical significance across immune subsets in AF; NK cells passed the FDR threshold. (c) Significant elevation of multiple immune cell types in ATH, including MAIT cells, iTregs, CD8^+^ naïve T cells, and T follicular helper (Tfh) cells. (d) ATH plot showing that 22 out of 24 immune subsets exhibited significant FDR-adjusted *p* values < 0.05 with MAIT cells and macrophages as the most altered populations.

Analysis using ImmuCellAI further elucidated these findings. In the AF dataset, the immune changes were limited, with only NK cells showing statistically significant elevation (*p* < 0.05) compared to controls (Figure 10a,b). Most other immune subsets, such as MAIT cells, effector memory T cells, and macrophages, showed distributional shifts that failed to reach statistical significance after FDR correction. In contrast, ATH samples demonstrated extensive immune remodeling according to ImmuCellAI (Figure 10c,d). Significant increases were observed in innate-like T cells (e.g., MAIT and *γδ* T cells), regulatory T cells (iTregs), CD8^+^ naïve T cells, and T follicular helper cells. Notably, 22 of 24 immune cell types reached FDR-adjusted *p* < 0.05, confirming widespread immune activation and regulation in atherosclerotic tissue. The strongest effects were detected in macrophage and MAIT cell populations (*p* < 0.0001), indicating an intense immune response profile. The results from CIBERSORTx and ImmuCellAI highlight both shared and distinct immune remodeling patterns in AF and ATH. While both diseases showed consistent dysregulation of macrophages and dendritic cells, AF was characterized by broader but subtler alterations, with NK cells and T helper subsets mildly perturbed. In contrast, ATH exhibited robust and widespread immune infiltration across multiple innate and adaptive lineages.

### 3.7. Single-Cell Transcriptomic and Cell–Cell Communication Analysis

**Figure 11:**
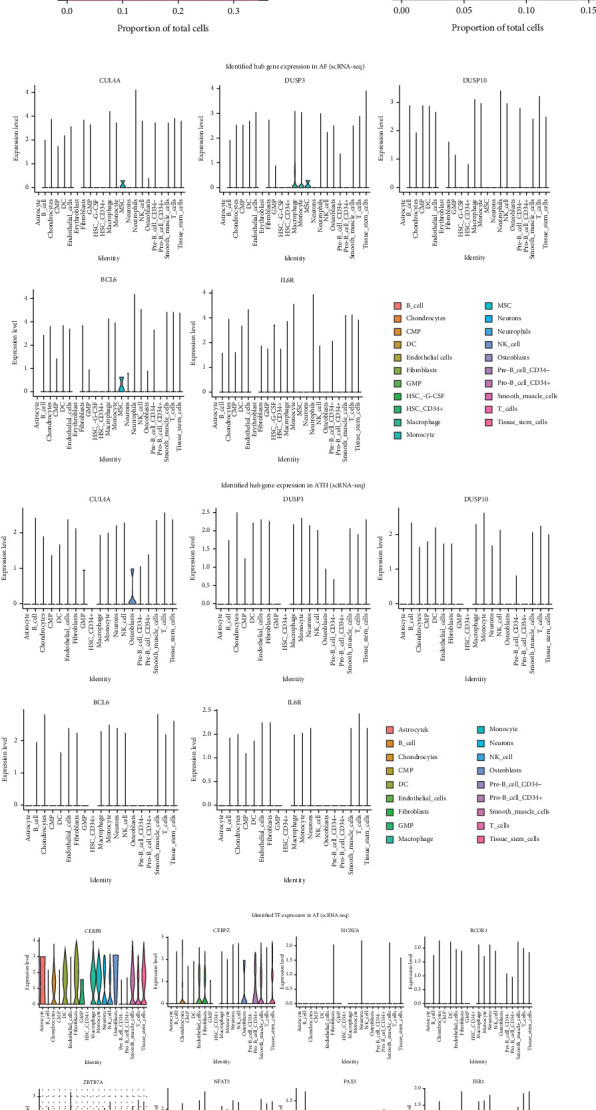
Single-cell RNA-seq analysis of cell populations and immune gene expression in AF and ATH. (a) UMAP projections of annotated cell types from scRNA-seq data of AF and ATH, labeled via SingleR with HumanPrimaryCellAtlas reference. (b) Horizontal bar plots of proportional cellular composition showing relative frequencies of endothelial cells, macrophages, fibroblasts, and lymphocytes. (c) Violin plots showing expression of five hub immune-related genes in cell type–specific resolution across AF (top) and ATH (bottom) tissues. (d) Violin plots displaying cell type–specific expression of transcription factors in AF (top) and ATH (bottom).

Dimensional reduction and cell type annotation using SingleR revealed distinct populations in both AF and ATH tissues (Figure 11a). ATH tissue showed prominent clusters of endothelial cells, macrophages, fibroblasts, and smooth muscle cells, whereas AF tissue exhibited enrichment in tissue-resident macrophages, NK cells, and dendritic cells. Relative cellular composition is visualized in horizontal bar plots (Figure 11b) with T cells and fibroblasts dominating the AF microenvironment and endothelial and macrophage subsets being more abundant in ATH. Expression profiling of the five LASSO-prioritized hub genes confirmed cell type–specific expression patterns. In AF, *BCL6* and *IL6R* were highly expressed in T cells and endothelial cells while *DUSP3* and *DUSP10* were enriched in macrophages and fibroblasts. In ATH, similar gene localization was observed (Figure 11c). Parallel analysis of nine TFs (Figure 11d) showed *CEBPZ*, *CEBPB*, and *HOXC6* predominantly expressed in myeloid-derived populations in both diseases, while *RCOR1*, *PAX5*, and *ZBTB7A* were distributed across T and endothelial cells. These expression patterns signify the relevance of these genes as regulatory mediators in tissue-specific immunity.

**Figure 12:**
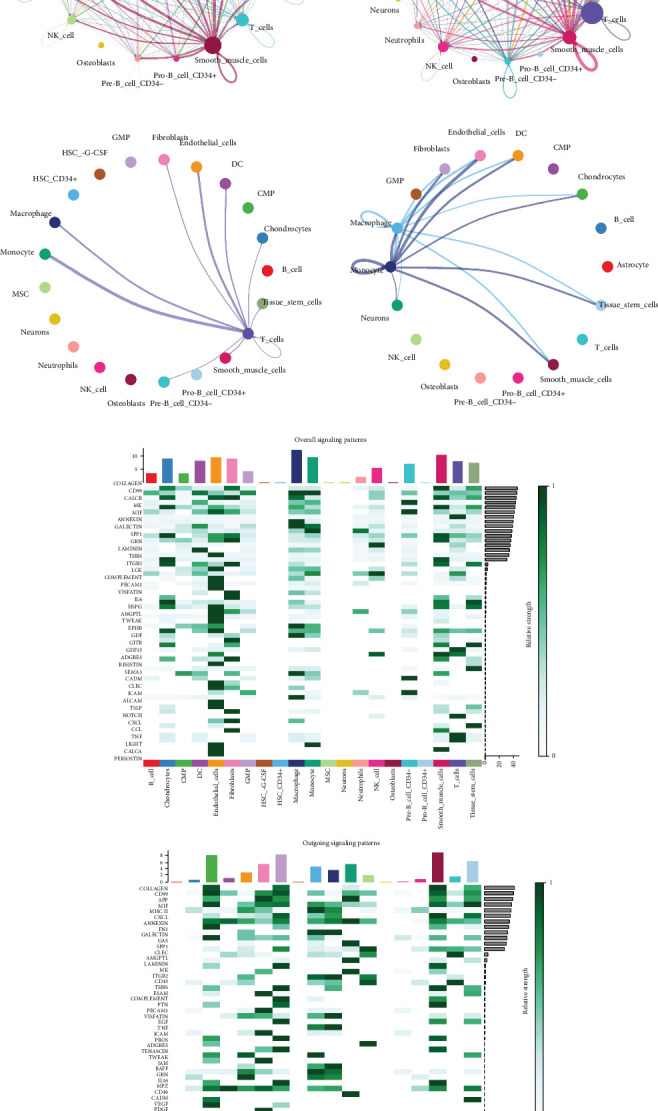
Intercellular communication analysis using CellChat in AF and ATH tissues. (a) Circle plots showing global ligand–receptor interaction networks among cell types in AF (right) and ATH (left). (b) Directed communication plots illustrating dominant signal-sending cell types in each condition. (c) Heatmaps of overall and outgoing signaling strength across cell types and pathways. Darker shades indicate stronger inferred interaction strength.

**Figure 13:**
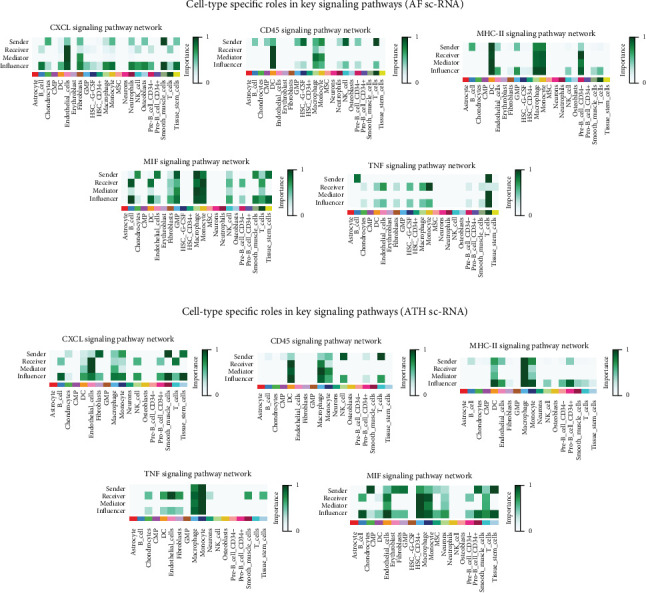
Cell type–specific signaling pathway roles in AF and ATH (scRNA-seq). (a) Heatmaps for five key signaling pathways in AF showing each cell type's role as sender, receiver, mediator, or influencer. (b) Analogous analysis in ATH revealing differences in pathway drivers across diseases. MHC-II signaling is enriched in dendritic cells in ATH, while *TNF* and *CXCL* signals in AF are predominantly mediated by fibroblasts and macrophages.

Intercellular communication modeling with CellChat revealed widespread ligand–receptor interactions across all cell types in both diseases (Figure 12a). Overall interaction density and connection strength appeared higher in AF, which suggests a more complex immunoregulatory microenvironment. Directed communication flow (Figure 12b) illustrated fibroblasts, smooth muscle cells, and macrophages as dominant signal senders in AF, while in ATH, endothelial and smooth muscle cells were key contributors to signaling outflow. Heatmaps summarizing overall and outgoing signal strengths (Figure 12c) identified high activity in *CXCL*, *IL1*, and *MIF* pathways. Further analysis of key immunoregulatory pathways including *CXCL*, *CD45*, *MHC-II*, *MIF*, and *TNF* revealed distinct cell type–specific roles. In AF (Figure 13a), fibroblasts and T cells were prominent senders in *CXCL* and *MIF* signaling, while macrophages acted as major mediators of TNF signals. In ATH (Figure 13b), MHC-II signaling was enriched in dendritic cells, and CD45 signaling was primarily mediated by endothelial cells and macrophages. These differential signaling maps indicate that, while both diseases share overlapping immunoregulatory modules, their cellular drivers and interaction strengths diverge significantly.

### 3.8. *BCL6*, *DUSP3*, *IL6R*, and *CUL4A* Regulate the Profibrotic Phenotype of Ang II-Treated MCFs

**Figure 14:**
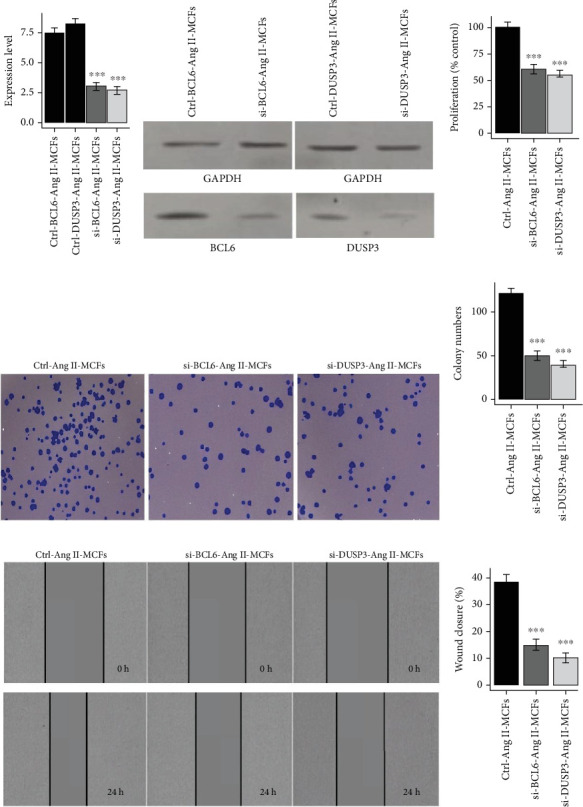
Silencing of *BCL6 and DUSP3* inhibits proliferation, colony formation, and migration in angiotensin II-treated mouse cardiac fibroblasts (Ang II-MCFs). (a) RT-qPCR analysis showing effective knockdown of *BCL6 and DUSP3* expression in siRNA-transfected Ang II-MCFs compared to control (Ctrl-Ang II-MCFs). (b) Western blot analysis further confirms the reduction of *BCL6 and DUSP3* protein levels posttransfection. (c) Cell proliferation was significantly decreased in both *si-BCL6 and si-DUSP3* groups, as shown by CCK-8 assay. (d, e) Colony formation assay revealed a substantial reduction in the number of colonies formed by *si-BCL6 and si-DUSP3* cells. (f, g) Wound healing assay demonstrated significantly impaired migration in *BCL6- and DUSP3-*deficient cells at 24 h compared to control.

**Figure 15:**
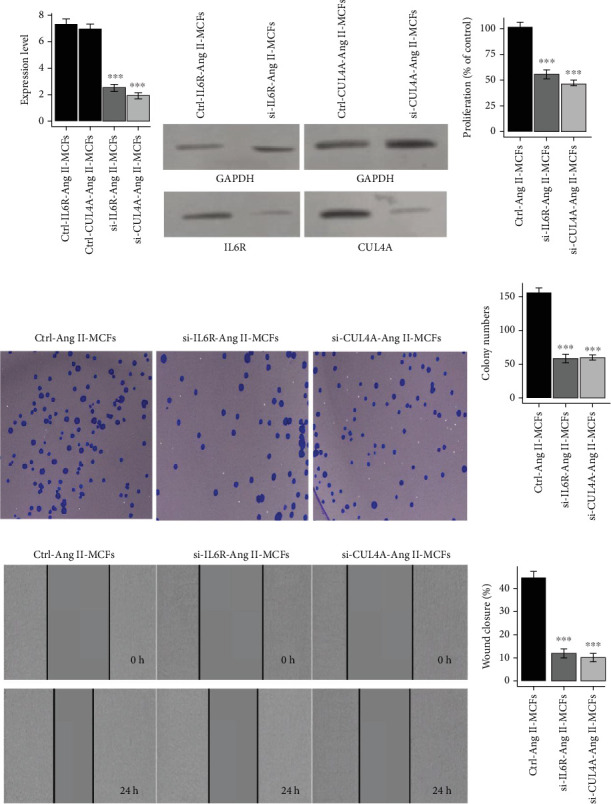
Functional knockdown of *IL6R and CUL4A* attenuates fibrotic characteristics in angiotensin II-treated mouse cardiac fibroblasts (Ang II-MCFs). (a) RT-qPCR analysis validating efficient suppression of *IL6R and CUL4A* transcript levels in siRNA-transfected Ang II-MCFs. (b) Corresponding western blot analysis confirms the knockdown of *IL6R and CUL4A* proteins. (c) CCK-8 assays show that the proliferation of Ang II-MCFs is significantly reduced upon *IL6R and CUL4A* silencing. (d, e) Colony formation ability was significantly decreased in cells lacking *IL6R or CUL4A*, as evidenced by fewer and smaller colonies. (f, g) Wound healing assays at 0 and 24 h demonstrate impaired migratory capacity in the *si-IL6R and si-CUL4A* groups relative to controls.

To experimentally validate the functional roles of key immune-related genes identified through integrative transcriptomic analysis of AF and ATH, we conducted in vitro loss-of-function assays in Ang II-treated mouse cardiac fibroblasts (Ang II-MCFs). These cells mimic the activated, profibrotic fibroblast phenotype commonly observed in AF pathology. RT-qPCR analysis confirmed robust knockdown of *BCL6*, *DUSP3*, *IL6R*, and *CUL4A* following siRNA transfection (Figures 14a and 15a), with corresponding reductions in protein expression validated by western blotting (Figures 14b and 15b), indicating efficient silencing at both transcript and protein levels. Functionally, gene silencing of *BCL6*, *DUSP3*, *IL6R*, or *CUL4A* led to a significant decrease in cell proliferation in transfected Ang II-MCFs compared to control cells (Figures 14c and 15c, *p* < 0.001). These findings were supported by colony formation assays, which demonstrated a marked reduction in the number of colonies formed upon knockdown of each gene (Figures 14e and 15e, *p* < 0.001), indicative of impaired clonogenic potential. In parallel, wound healing assays revealed that knockdown of all four genes significantly suppressed fibroblast migration, as demonstrated by reduced wound closure after 24 h (Figures 14g and 15g, *p* < 0.001), further supporting a role in maintaining the migratory capacity of profibrotic fibroblasts.

## 4. Discussion

In this study, we employed a comprehensive system-level approach integrating bulk transcriptomics, immune deconvolution, and single-cell RNA sequencing to investigate shared immune-regulatory mechanisms in AF and ATH. By performing differential gene expression analysis on two independent datasets per disease, we identified a robust set of 29 overlapping DEGs between AF and ATH. Functional annotation and enrichment analyses revealed consistent dysregulation of immune and inflammatory pathways with strong involvement of phosphatase activity, MAPK signaling, and T cell-mediated modulation. From within this intersecting gene set, five IDEGs were identified, that is, *BCL6*, *DUSP3*, *IL6R*, *CUL4A*, and *DUSP10*, which reflect convergence on cytokine regulation, phosphatase signaling, and immune resolution. LASSO regression modeling further prioritized *BCL6*, *DUSP3*, and *IL6R* as the most discriminatory features. Subsequent validation of these IDEGs and their associated nine transcriptional factors in external microarray datasets confirmed consistent expression trends of these genes.

The findings of this study offer novel insights into the overlapping immunopathological landscape of AF and ATH. Both diseases are increasingly recognized to involve chronic low-grade inflammation, but the shared molecular underpinnings have remained incompletely defined. Our data indicate that immune gene reprogramming, particularly involving phosphatase-related genes such as *DUSP3* and *DUSP10* and cytokine receptors like *IL6R*, may serve as common hubs linking atrial and vascular pathology. This aligns with previous literature implicating the IL-6 axis in vascular inflammation and atrial remodeling [51] and BCL6's role in repressing inflammatory responses and T cell differentiation [52]. The identification of *BCL6* as a hub transcriptional repressor across both diseases suggests potential for shared epigenetic or regulatory feedback loops in cardiovascular immune dysregulation. Pathway enrichment analyses further confirmed a unified functional axis between AF and ATH. GO terms such as “MAP kinase phosphatase activity” and “negative regulation of the JNK/ERK cascade” reflect disruptions in canonical signaling checkpoints that regulate both inflammatory and fibrotic pathways. Enriched KEGG and Reactome pathways showed the convergence of metabolic and cytokine-based remodeling. The consistent enrichment of these terms across multiple annotation databases reinforces the biological coherence of the identified genes. Moreover, disease–gene association mapping linked several DEGs to cardiovascular comorbidities such as heart failure, diabetic retinopathy, and pulmonary hypertension, which suggests broader pathophysiological relevance.

While the LASSO model refined the immune gene list to three classifier genes, downstream functional and regulatory analyses were performed using all five initially intersecting IDEGs due to the biological importance of *CUL4A* and *DUSP10*, both of which were enriched in immunomodulatory pathways [53, 54] and exhibited context-specific expression in validation datasets. Drug–target interaction analysis revealed that four of the five IDEGs are considered druggable, with *IL6R* already targeted clinically by agents such as tocilizumab [55]. This translational aspect is particularly relevant given the emerging interest in immune modulation for cardiovascular disease. *BCL6* inhibitors are under preclinical development for oncologic and inflammatory disorders [56] while dual-specificity phosphatase inhibitors targeting *DUSP3* may offer a novel therapeutic angle, albeit with limited current pharmacological tools [57]. These findings provide a rational starting point for compound prioritization and potential repurposing of immune-targeted therapies for cardiovascular comorbidities. TF analysis using iRegulon identified nine upstream regulators with high NESs, including *SCRT2*, *CEBPZ*, *HOXC6*, *NFAT5*, and *ZBTB7A*. These TFs collectively converged on *BCL6*, *IL6R*, and *DUSP3*, suggesting transcriptional coordination of shared immune responses in AF and ATH. While several of these TFs have been implicated in immune modulation, vascular remodeling, or cardiac development, their coregulation of this gene set presents a compelling hypothesis for future mechanistic studies. Expression validation across independent datasets revealed context-specific upregulation patterns for *RCOR1*, *ZBTB7A*, and *NFAT5*, supporting their candidacy as upstream disease modulators as documented by several studies [58–60]. Notably, *SCRT2*, despite being identified as a TF through iRegulon analysis (NES = 5.73), was not detected in either the external validation microarray datasets or the sc-RNA seq profiles, which indicates its possible tissue-specific expression limitation or platform-dependent detection sensitivity.

Immune cell deconvolution analysis using both CIBERSORTx and ImmuCellAI highlighted distinct immune remodeling patterns in AF and ATH. While macrophage subsets, particularly M0 macrophages, were consistently enriched in both diseases, AF displayed broader but subtler changes across T cell and dendritic compartments. In contrast, ATH was marked by a widespread and statistically significant elevation in innate-like T cells (e.g., MAIT cells), regulatory T cells, and macrophages. This divergence shows disease-specific immune topographies; that is, AF appears to reflect a mixed immune environment with functional dysregulation of both innate and adaptive components, whereas ATH is characterized by expansive immune infiltration dominated by myeloid and innate-like lymphocyte subsets. Finally, single-cell transcriptomic analysis further refined these findings by localizing key gene expression and intercellular signaling events to specific cell types. CellChat modeling revealed increased *CXCL*, *MIF*, and *TNF* signaling in AF, primarily mediated by fibroblasts, macrophages, and T cells, while ATH showed stronger *MHC-II* and *CD45*-mediated interactions driven by endothelial and dendritic cells. This cell-level resolution highlights divergent immunoregulatory hierarchies in each disease and supports the presence of shared, yet mechanistically distinct, immune–stromal signaling circuits. In summary, our multilayered analysis identifies *BCL6*, *DUSP3*, and *IL6R* as robust cross-disease immune signatures in AF and ATH, with evidence for shared regulatory, transcriptional, and intercellular signaling mechanisms. Future studies should prioritize experimental validation of these hub genes and TFs to define their causal roles and therapeutic tractability in immune-mediated cardiovascular disease.

## 5. Conclusion

This study presents a systems immunology analysis that integrates bulk transcriptomics, immune cell deconvolution, drug–gene mapping, and single-cell RNA sequencing to uncover convergent molecular mechanisms in AF and ATH. We identified 29 overlapping DEGs between AF and ATH datasets and prioritized five immune-related genes using curated immune gene sets. Among these, *BCL6*, *DUSP3*, and *IL6R* were selected by LASSO regression as robust diagnostic features. Downstream functional analyses revealed biologically coherent enrichment in MAPK phosphatase activity, IL-6 signaling, ERK cascade modulation, and T cell differentiation. Drug–target interaction analysis confirmed that four of the five genes are druggable, with *IL6R* and *BCL6* already associated with clinical or investigational therapies. Regulatory network modeling uncovered nine TFs that coregulate the prioritized hub genes. These transcriptional regulators were validated at the expression level and demonstrated context-specific modulation across diseases. Immune infiltration profiling by CIBERSORTx and ImmuCellAI illustrated shared myeloid activation, particularly involving macrophage subsets. Single-cell RNA-seq analysis confirmed cell type–specific expression of hub genes and TFs and revealed contrasting intercellular signaling topologies using CellChat. This work provides a unified framework for exploring immune axes across comorbid cardiovascular conditions and lays the foundation for future mechanistic validation.

## Figures and Tables

**Table 1:** Summary of transcriptomic datasets used for differential gene expression analysis and single-cell validation in AF and ATH.

**Disease**	**GEO series**	**GPL**	**Group type**	**Cases**	**Controls**	**Total arrays**	**Notes**
Atrial fibrillation	GSE115574	GPL570	Training	30⁣^∗^	29⁣^∗^	59	Left/right atrium paired samples
Atrial fibrillation	GSE41177	GPL570	Training	32	6	38	Atrial tissue: Persistent AF vs. sinus rhythm
Atrial fibrillation	GSE79768	GPL570	Validation/test	14⁣^∗∗^	12⁣^∗∗^	26	Paired left/right atrium, SR vs. persistent AF
Atrial fibrillation	GSE14975	GPL570	Validation/test	5	5	10	Left atrial tissue biopsies
Atherosclerosis	GSE100927	GPL17077	Training	69	35	104	Carotid, femoral, infrapopliteal artery samples
Atherosclerosis	GSE57691	GPL10558	Training	58	10	68	Abdominal aortic aneurysm/occlusive disease vs. donor aorta
Atherosclerosis	GSE41571	GPL570	Validation/test	5	6	11	Macrophage-rich regions in ruptured vs. stable carotid plaques
Atherosclerosis	GSE13985	GPL570	Validation/test	5	5	10	Familial hypercholesterolemia vs. healthy in blood
Atherosclerosis	GSE159677	10× Genomics Chromium	scRNA-seq	6	—	6	Coronary artery plaques vs. adjacent normal tissue
Atrial fibrillation	GSE255612	10× Genomics Chromium	scRNA-seq	18	16	34	Atrial appendage single-cell RNA from AF and SR patients

*Note*: Asterisk (∗) indicates single atrial tissue counts; double asterisks (∗∗) indicate paired left and right atrial tissue counts, all other datasets include one sample per patient.

**Table 2:** Top enriched GO biological processes (nominal *p* < 0.01; nonsignificant after FDR correction).

**Term**	**p** ** value**	**q** ** value**	**Overlap genes**
Positive regulation of regulatory T cell differentiation (GO:0045591)	0.000272	0.072574	DUSP10, BCL6
Regulation of regulatory T cell differentiation (GO:0045589)	0.000504	0.072574	DUSP10, IL6R
Negative regulation of JNK cascade (GO:0046329)	0.001042	0.100070	DUSP3, DUSP10
Negative regulation of ERK1 and ERK2 cascade (GO:0070373)	0.002382	0.105091	DUSP3, DUSP10
Positive regulation of T cell differentiation (GO:0045582)	0.003871	0.105091	DUSP10, BCL6
Positive regulation of focal adhesion disassembly (GO:0120183)	0.007230	0.105091	DUSP3
Intracellular phosphate ion homeostasis (GO:0030643)	0.007230	0.105091	XPR1
Glycosyl compound biosynthetic process (GO:1901659)	0.007230	0.105091	TYW3
Wybutosine metabolic process (GO:0031590)	0.007230	0.105091	TYW3
Wybutosine biosynthetic process (GO:0031591)	0.007230	0.105091	TYW3

## Data Availability

All datasets analyzed in this study can be accessed through the NCBI GEO portal at https://www.ncbi.nlm.nih.gov/geo/. The microarray datasets used for AF include GSE115574, GSE41177, GSE79768, and GSE14975. ATH datasets include GSE100927, GSE57691, GSE41571, and GSE13985. Single-cell RNA sequencing data for AF and ATH were retrieved from GSE159677 and GSE131778, respectively. The custom R scripts are available from the corresponding authors upon reasonable request.
